# Single-cell transcriptome analysis profiles the expression features of TMEM173 in BM cells of high-risk B-cell acute lymphoblastic leukemia

**DOI:** 10.1186/s12885-023-10830-5

**Published:** 2023-04-24

**Authors:** Yiqing Cai, Xiaomin Chen, Tiange Lu, Zhuoya Yu, Shunfeng Hu, Jiarui Liu, Xiangxiang Zhou, Xin Wang

**Affiliations:** 1 Department of Hematology, Shandong Provincial Hospital, Shandong University, No.324, Jingwu Road, Jinan, Shandong 250021 China; 2 Department of Hematology, Shandong Provincial Hospital Affiliated to Shandong First Medical University, No.324, Jingwu Road, Jinan, Shandong 250021 China; 3 Shandong Provincial Engineering Research Center of Lymphoma, Jinan, Shandong 250021 China; 4 Branch of National Clinical Research Center for Hematologic Diseases, Jinan, Shandong 250021 China; 5 National Clinical Research Center for Hematologic Diseases, the First Affiliated Hospital of Soochow University, Suzhou, 251006 China

**Keywords:** B-cell acute lymphoblastic leukemia, Single-cell RNA-sequencing, TMEM173, Immune cells

## Abstract

**Background:**

As an essential regulator of type I interferon (IFN) response, TMEM173 participates in immune regulation and cell death induction. In recent studies, activation of TMEM173 has been regarded as a promising strategy for cancer immunotherapy. However, transcriptomic features of TMEM173 in B-cell acute lymphoblastic leukemia (B-ALL) remain elusive.

**Methods:**

Quantitative real-time PCR (qRT-PCR) and western blotting (WB) were applied to determine the mRNA and protein levels of TMEM173 in peripheral blood mononuclear cells (PBMCs). TMEM173 mutation status was assessed by Sanger sequencing. Single-cell RNA sequencing (scRNA-seq) analysis was performed to explore the expression of TMEM173 in different types of bone marrow (BM) cells.

**Results:**

The mRNA and protein levels of TMEM173 were increased in PBMCs from B-ALL patients. Besides, frameshift mutation was presented in TMEM173 sequences of 2 B-ALL patients. ScRNA-seq analysis identified the specific transcriptome profiles of TMEM173 in the BM of high-risk B-ALL patients. Specifically, expression levels of TMEM173 in granulocytes, progenitor cells, mast cells, and plasmacytoid dendritic cells (pDCs) were higher than that in B cells, T cells, natural killer (NK) cells, and dendritic cells (DCs). Subset analysis further revealed that TMEM173 and pyroptosis effector gasdermin D (GSDMD) restrained in precursor-B (pre-B) cells with proliferative features, which expressed nuclear factor kappa-B (NF-κB), CD19, and Bruton’s tyrosine kinase (BTK) during the progression of B-ALL. In addition, TMEM173 was associated with the functional activation of NK cells and DCs in B-ALL.

**Conclusions:**

Our findings provide insights into the transcriptomic features of TMEM173 in the BM of high-risk B-ALL patients. Targeted activation of TMEM173 in specific cells might provide new therapeutic strategies for B-ALL patients.

**Supplementary Information:**

The online version contains supplementary material available at 10.1186/s12885-023-10830-5.

## Introduction

B-cell acute lymphoblastic leukemia (B-ALL) is an aggressive type of acute leukemia characterized by the clonal expansion of immature B cells and extensive extramedullary infiltration [1]. B-ALL patients cover different age groups and genders, but clinical outcomes vary from children to adults [2]. In general, the prognosis of adult patients is far worse than that of children [2]. Lower treatment responses of adult patients are attributed to the heterogenic transcriptome, pre-existing comorbidities, and poor tolerance of chemotherapies [3, 4]. Although remission rates are appreciable, long-term maintenance of complete remission (CR) can still be ruined by bone marrow (BM) recurrence [5]. Given the special situation, treatment regimens for refractory/relapse (R/R) B-ALL remain undetermined [6, 7]. Recently, targeting tumor immunity has been regarded as a critical approach to improving therapeutic efficacy [8, 9]. Based on the capability of regulating immune cell activation, chimeric antigen receptor T (CAR-T) cells and immune checkpoint inhibitors have achieved impressive efficacy in patients with R/R B-ALL [10, 11]. Therefore, molecular mechanisms of tumor immunoregulation associated with tumorigenesis and progression deserve in-depth exploration.

Tissue-specific tumor microenvironment (TME) is a crucial factor in anti-tumor immune response [12, 13]. Accumulating evidence has considered the stimulator of interferon genes (STING, also named TMEM173) as a vital regulator in immunoregulation [14, 15]. TMEM173 has a well-known role in innate immunity via regulating immune cells and signaling pathways [16, 17]. On the one hand, TMEM173 activation contributes to establishing crosstalk between tumor cells and TME cells, further leading to TME remodeling [18] and anti-tumor response [19–21]. Hence, TMEM173 agonists have been approved for several pre-clinical models and clinical trials [22], especially in patients with advanced and refractory tumors [23–25]. On the other hand, TMEM173 has been reported to negatively regulate B cell receptor (BCR) signaling in both normal and malignant B cells [26]. At present, the biological function of TMEM173 in high-risk B-ALL remains unknown.

Increasing studies highlight the importance of transcriptional heterogeneity in tumorigenesis, which is associated with functional changes in cell viability, distant metastasis, and drug resistance [27, 28]. Identification of cell-specific genes and TME features provides insights into the mechanisms of tumorigenesis and tumor progression [29, 30]. Single-cell RNA sequencing (scRNA-seq) is a new technology of high-throughput sequencing analysis, which can be used to interrogate features of tumor tissues at a single-cell resolution [31, 32]. In this study, scRNA-seq analysis was performed to investigate the transcriptomic features of TMEM173 in high-risk B-ALL patients, which profiled the differential expression of TMEM173 in BM cells. Specifically, proliferated precursor-B (pre-B) cells, cytotoxic natural killer (NK) cells, and activated dendritic cells (DCs) expressed low levels of TMEM173. This characterization facilitated understanding biological functions of TMEM173 and enabled the identification of new targets for immunotherapy.

## Methods

### Peripheral blood samples

Peripheral blood samples were obtained from 6 newly diagnosed B-ALL patients and 3 healthy donors in Shandong Provincial Hospital. Clinical data of enrolled patients and healthy donors were presented in Table 1. The diagnostic criteria were established according to the World Health Organization (WHO) classification [33]. Peripheral blood mononuclear cells (PBMCs) were isolated using the Ficoll-Hypaque density gradient centrifugation method (TBD Science, China) [34]. PBMCs of 3 healthy donors were labeled by Normal (N) while 6 patient samples were labeled by Tumor (T). In accordance with the Helsinki declaration, the acquisition of clinical samples complied with the informed consent. This study was approved by the Medical Ethical Committee of Shandong Provincial Hospital.

**Table 1:** Clinical features of 6 B-ALL patients and 3 healthy donors

Number	Sex	Age (years)	Diagnosis	Mutant genes	Diagnosis karyotypes	White blood cell counts (1 × 10^9/L)	Percentage of Naive cells in BM (%)	Stage
T1	Female	49	B-ALL	TP53, ZEB1, BCORL1, SPEN, KLF2, FAT1, FOXO3, KMT2C	47, XX, + 1 der(1;16)(q10;p10), + 8, t(9;22)(q34;q11.2) [3]	134.13	94	Diagnosis
T2	Male	15	B-ALL	ETV6, KRAS, EZH2	-	21.48	93	Diagnosis
T3	Male	67	Pre-B ALL	KRAS, NF1, PTEN, DNM2, TCF3, TP53	73 ~ 79 < 3n>,XXY, + 1, +4,+6, + 8, add(9)(p22); +16, + 17, -17, + 19, +21, + 22, inc [cp4]	5.69	99	Diagnosis
T4	Female	56	B-ALL (High-risk)	KRAS, NF1, PTEN, DNM2, TCF3, TP53	46,XX,t(2;12)(p13;p11),del(8)(p21) [6] /46,XX [4]	17.17	95	Diagnosis
T5	Male	46	Ph-positive B-ALL (High-risk)	BCR-ABL1	46, XY, t(9;22)( (q34;q11) [10]	52.69	90	Diagnosis
T6	Female	64	Ph-positive B-ALL (High-risk)	BCR-ABL1, SF1, USP7, SPIB, PLCG1, SF3A1, EP300, POT1, PTPRD	46, XX, t(9;22)( (q34;q11) [2]	57.35	96	Diagnosis
N1	Female	30						
N2	Male	25						
N3	Male	27						

### Quantitative real-time PCR (qRT-PCR)

Total RNA was extracted from each PBMC sample (N = 3, T = 6) by the TRIzol reagent (TaKaRa, 9108-1, Japan). After removing the genomic DNA from RNA templates, RNA was reverse transcribed into cDNA using HiScript III RT SuperMix (Vazyme, R323-01, China). Amplification reactions were performed in Light Cycler 480II (Roche) with the FastStart™ Universal SYBR Green (Roche, Switzerland) [35, 36]. Reactions were carried out for 30 s at 95°C, followed by 50 cycles of PCR for 10 s at 95 ℃, 20 s at 55 ℃, and 20 s at 72 ℃. Data were evaluated using the 2^−ΔΔCt^ method. Primers of TMEM173: Forward, 5’-TACAACAACCTGCTACGGGG-3’; Reverse, 5’-TCTGCTGGGGCAGTTTATCC-3’. Primers of GAPDH: Forward, 5’-GGTGAAGGTCGGAGTCAACG-3’; Reverse, 5’-CAAAGTTGTCATCGAATGAC-3’. Primers of β-actin: Forward, 5’-CATGTACGTTGCTATCCAGGC-3’; Reverse, 5’-CTCCTTAATGTCACGCACGAT-3’.

### Sanger sequencing

Total RNA was extracted from PBMCs of B-ALL (T = 6), followed by genomic DNA wiper and reverse transcription. Sanger sequencing was performed by the BioSune Technology Co., Ltd. (Shanghai, China) according to the manufacturer’s protocol.

### Western blotting (WB)

WB was performed as previously described [37, 38]. Briefly, cell lysates of each PBMC sample (N = 3, T = 6) were resolved by 10% SDS-PAGE and transferred onto polyvinylidene fluoride (PVDF) membranes. Full-length PVDF membranes were blocked in 10% milk in Tris-buffered saline and Tween 20 (TBST), and incubated with primary antibodies (1:1000) overnight at 4℃. Nitrocellulose-bound primary antibodies were combined with HRP-linked secondary antibodies (1:5000). Chemiluminescent signals were detected by the Amersham Imager 680 (GE Healthcare, UK). After performing 3 replicate experiments, expression levels of protein were represented by gray values and calculated by the Image J software (National Institutes of Health, USA). Primary antibodies were listed as follows: anti-rabbit TMEM173 (Cell Signaling Technology, 13647, USA) and anti-mouse GAPDH (Zhongshan Gold Bridge, TA-08, China).

### Linear dimensionality reduction and cell clustering

ScRNA-seq data of 2 healthy donors and 2 high-risk B-ALL patients was obtained from the Gene Expression Omnibus (GEO) database (GSE130116, **Supplementary Table 1**). ScRNA-seq data of each patient included BM cells at the time of initial diagnosis and relapse (**Supplementary Table 1**). Original data was loaded into Seurat version 3.6.3 and processed by the Seurat package. Firstly, scRNA-seq data was fully merged by the Harmony package, followed by data filtering, normalization, and removal of batch effects. For data filtering, genes detected in less than 3 cells or with counts of zero across all cells were excluded. Data normalization was then conducted by the NormalizeData function and the global-scaling log-normalization with a scale factor of 10,000. Subsequently, 4,000 high-variable genes were extracted using the FindVariableGenes function, followed by the identification of principal component analysis (PCA) and principal components (PCs). Based on the ElbowPlot function, the top 50 PCs were screened for cell clustering. Finally, t-distributed stochastic neighbor embedding (t-SNE) was applied to realize cell clustering.

### Cell type annotation and genes expression analysis

Cell type annotation was conducted by a combination of the FindAllMarkers function and the CellMarker (http://biocc.hrbmu.edu.cn/CellMarker/index.jsp). Marker genes of each cluster were extracted by the FindAllMarkers function and subsequently introduced into the CellMarker to distinguish cell types. The typical marker genes of each cell cluster were listed in **Supplementary Tables 1** and exhibited by heatmap plots. The FeaturePlot, VlnPlot, DotPlot, and RidgePlot functions were used to analyze gene expression features. Besides, differentially expressed genes (DEGs) were illustrated using volcano plots. Finally, pseudo-time analysis performed by the Monocle package was applied to display cell development trajectory and gene expression features over time.

### Statistical analysis

All data are recorded using mean ± standard deviation (SD) from at least three separate experiments. The GraphPad Prism software (v8.0a, La Jolla, CA, USA) was applied to the statistical analysis. The relative expression of mRNA and the grayscale values of protein were confirmed to be normally distributed. The statistical significance between the two groups was thereby determined by the unpaired two-tailed T-test with assumed normal distribution. P < 0.05 was considered statistically significant. ^*^P < 0.05, ^**^P < 0.01.

## Results

### TMEM173 was differentially expressed in BM cells of high-risk B-ALL patients

**Fig. 1:**
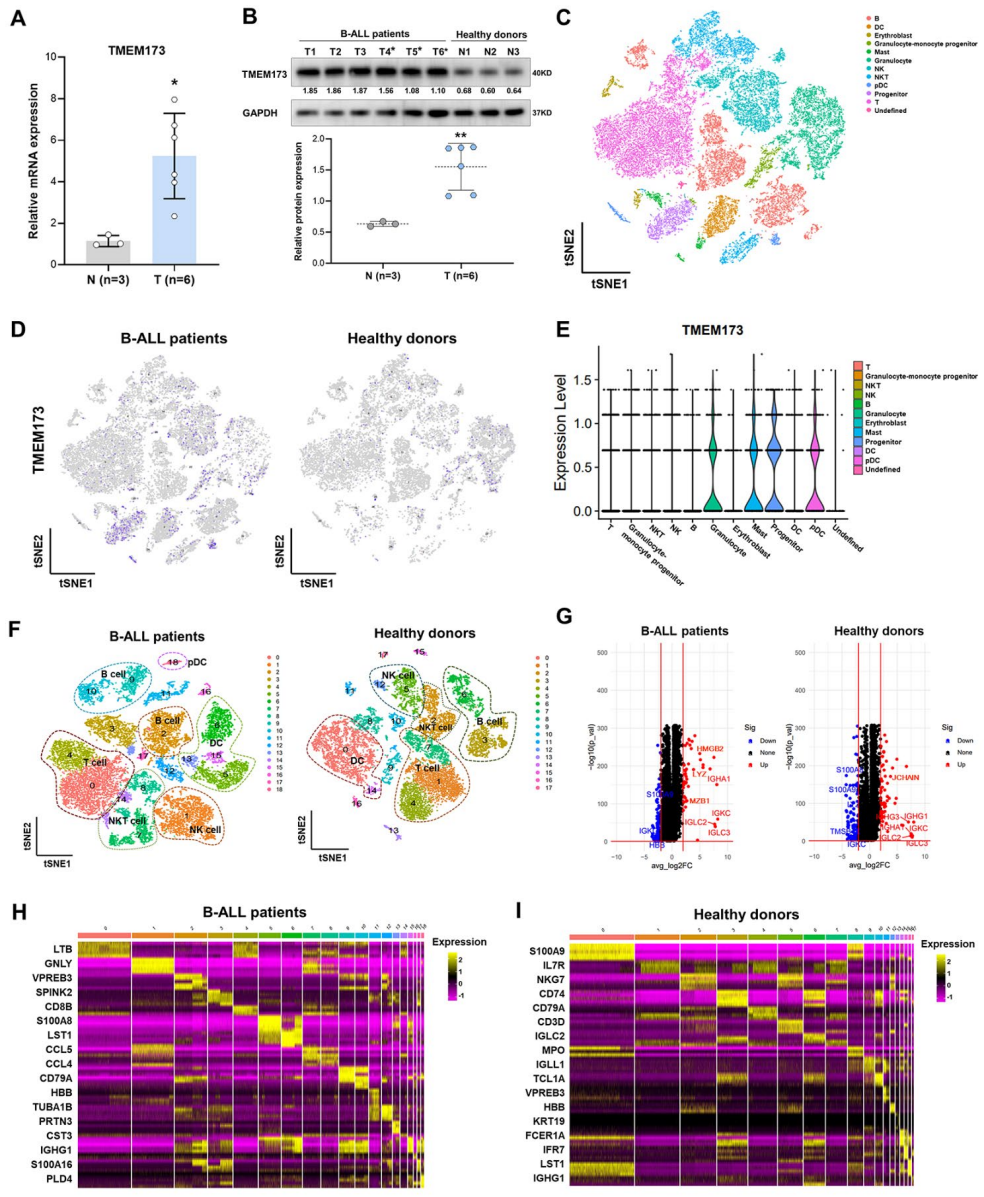
The transcriptome profiles of BM cells in B-ALL patients were different from healthy donors. **A, B**. Total RNA and protein were extracted from PBMCs of each individual (N1-N3 = healthy donors; T1-T6 = B-ALL patients). (**A**) Relative mRNA levels of TMEM173 were increased in PBMCs from 6 B-ALL patients (p < 0.05, Reference gene = GAPDH). (**B**) Quantitative (upper) and relative levels (lower) of TMEM173 protein in PBMCs of 6 B-ALL patients (p < 0.05, Reference gene = GAPDH). 3 asterisk-marked samples (T4-T6) were obtained from high-risk B-ALL patients. Representative images were extracted from the full-length PVDF membranes around the molecular weight of TMEM173 and GAPDH. The original images were presented in **Supplementary Fig. 6**. **C-E**. ScRNA-seq data of 2 healthy donors and 2 B-ALL patients (both diagnosis and recurrence) was fully merged by the Harmony package. (**C**) Cell clustering with cell type annotation identified cell compositions in BM cells. (**D**) Differential expression of TMEM173 between B-ALL patients and healthy controls was profiled in t-SNE plots by using the Split-by function. (**E**) Violin plot compared the expression levels of TMEM173 in different types of BM cells. **F-I.** BM cells from B-ALL patients (both diagnosis and recurrence) and healthy donors were merged separately. (**F**). Cell clustering with cell type annotation displayed cell compositions in B-ALL (left) and healthy BM (right). (**G**) Volcano plots compared the DEGs between B cells and all others in B-ALL (left) and healthy BM (right). Heatmap analysis exhibited the most significant DEGs of each cell cluster in B-ALL patients (**H**) and healthy donors (**I**). Vertical bars in **A and B** indicated mean ± SD from three separate experiments. The statistical significance was determined by the unpaired two-tailed T-test. ^*^p < 0.05, ^**^p < 0.01.

We first investigated the expression and mutations of TMEM173 in PBMCs of B-ALL patients. The results showed that the mRNA and protein levels of TMEM173 were significantly increased in PBMCs of B-ALL patients (Fig. 1A, B; **Supplementary Fig. 1A**). Furthermore, frameshift mutation in TMEM173 sequences was detected in 2 B-ALL patients, accompanied by the other somatic mutations in B-ALL patients (Tables 1 and 2; **Supplementary Fig. 1B**).

**Table 2:** TMEM173 sequences in PBMC samples of B-ALL patients

Number	Diagnosis	TMEM173 sequence
T1	B-ALL	TCTCCTCCATT GGACTGTGGG GTGCCTGATA ACCTGAGTAT GGCTGACCCC AACATTCGCT TCCTGGATAA ACTGCCCCAG CAGAA
T2	B-ALL	TCTCCTCCATT GGACTGTGGG GTGCCTGATA ACCTGAGTAT GGCTGACCCC AACATTCGCT TCCTGGATAA ACTGCCCCAG CAGA
T3	Pre-B ALL	TCTCCTCCCAT TGGACTGTGG GGTGCCTGAT AACCTGAGTA TGGCTGACCC CAACATTCGC TTCCTGGATA AACTGCCCCA GCAGAA
T4	B-ALL (High-risk)	TCTCCTCCATT GGACTGTGGG GTGCCTGATA ACCTGAGTAT GGCTGACCCC AACATTCGCT TCCTGGATAA ACTGCCCCAG CAGAA
T5	Ph-positive B-ALL (High-risk)	TCTCCTCCATT GGACTGTGGG GTGCCTGATA ACCTGAGTAT GGCTGACCCC AACATTCGC TTCCTGGATAA ACTGCCCCAG CAGAA
T6	Ph-positive B-ALL (High-risk)	TCTCCTCCCAT TGGACTGTGG GGTGCCTGAT AACCTGAGTA TGGCTGACCC CAACATTCGCT TCCTGGATAA ACTGCCCCAG CAGAA

Given that PBMCs contained multiple types of cells, expression features of TMEM173 in PBMCs could not fully explain its expression changes in the leukemia-associated microenvironment. Hence, scRNA-seq analysis was performed to explore the transcriptome atlas of BM cells in high-risk B-ALL patients. To obtain detailed profiling of the B-ALL-associated microenvironment, original BM samples were separated into CD19^+^ B cell and CD19^−^CD45^+^ non-B cell proportions and mixed at a ratio of 1:5 [39]. After incorporating the BM cells of healthy donors and B-ALL patients, 30 transcriptionally-distinct cell clusters were identified by unbiased clustering (**Supplementary Fig. 2A**). As expected, cell compositions in BM varied from healthy donors to B-ALL patients (**Supplementary Fig. 2B)**. Based on the expression of typical marker genes, BM cells were classified into B cells, T cells, DCs, NK cells, NKT cells, monocytes, mast cells, erythroblasts, and progenitor cells (**Supplementary Fig. 2C**, Fig. 1C). T-SNE and violin plots further revealed the transcriptome features of TMEM173. Expression levels of TMEM173 were increased in granulocytes, progenitor cells, mast cells, and plasmacytoid dendritic cells (pDCs), especially in the BM of B-ALL patients (Fig. 1D, E). On the contrary, B cells, T cells, NK cells, and DCs were featured with low levels of TMEM173 (Fig. 1D, E). The above findings revealed the differential expression of TMEM173 in BM cells of high-risk B-ALL patients.

### Cellular composition and transcriptomic signatures of BM cells from B-ALL patients were different from that of healthy donors

The differential expression of TMEM173 in BM cells prompted us to investigate whether the cellular compositions and transcriptomic features differed between high-risk B-ALL patients and healthy donors. We first explored the differences in cell composition. Cell clustering profiled 19 clusters in B-ALL patients and 18 clusters in healthy donors **(**Fig. 1F**).** Based on the specific marker genes, BM cells were classified into different types, mainly including B cells, T cells, NK cells, NKT cells, and DCs (**Supplementary Table 2**, Fig. 1F**)**. In particular, B cells, NKT cells, and DCs were divided into more clusters in B-ALL patients, suggesting the changes in cell composition in the BM of B-ALL (Fig. 1F).

Since B-ALL originates from B lymphocytes, we compared the transcriptional differences between B cells and all other cells by volcano plots. As shown in Fig. 1G, B cell populations from B-ALL patients significantly elevated the expression of immunoglobulin heavy constant alpha-1 (IGHA1), immunoglobulin kappa constant (IGKC), immunoglobulin lambda constant 2 (IGLC2), immunoglobulin lambda constant 3 (IGLC3), marginal zone B and B-1 cell-specific protein (MZB1), high mobility group box 2 (HMGB2), and lysozyme (LYZ). DEGs of B cells from healthy donors were different from B-ALL patients, represented by up-regulated immunoglobulin heavy constant gamma-1 (IGHG1), immunoglobulin heavy constant gamma-3 (IGHG3), and joining chain of multimeric IgA and IgM (JCHAIN) **(**Fig. 1G**)**. It was worth noting that up-regulated DEGs were associated with B cell proliferation and immunoglobulin activity [40–42], indicating the functional alterations of B cells in B-ALL. Heatmap further exhibited the heterogeneous DEGs of different cell components in B-ALL patients and healthy donors (Fig. 1H, I). The above results indicate the changes in cell compositions and transcriptome profiles of B-ALL.

### Transcriptome features of TMEM173 and GSDMD in B cells

#### Leukemic cells of B-ALL patients reduced the expression of TMEM173 and GSDMD

**Fig. 2:**
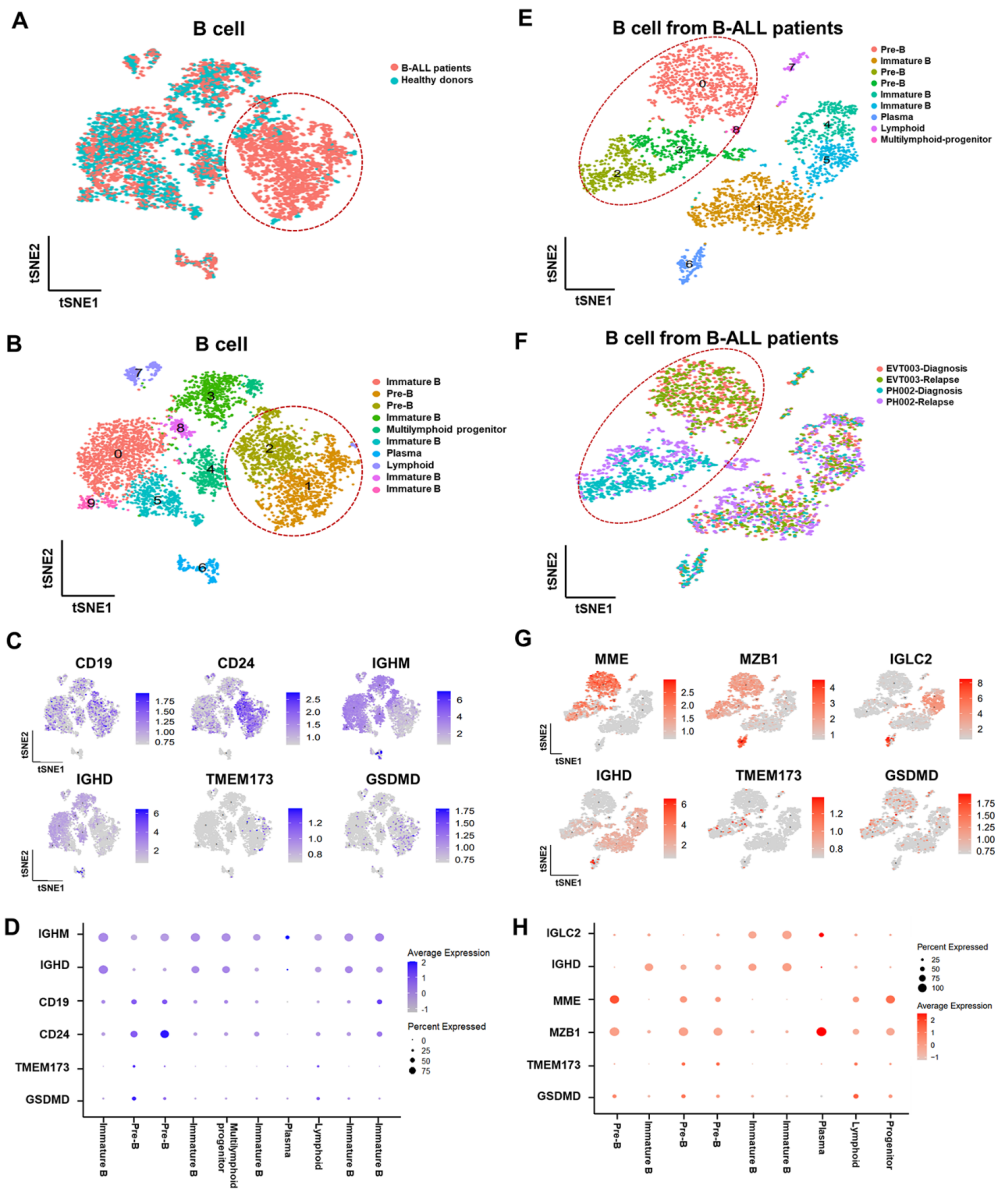
Expression levels of TMEM173 and GSDMD were decreased in pre-B cell clusters. **A-D.** Transcriptome features of TMEM173 in BM B cells. B cell clusters were extracted from total BM cells. (**A**) Cell clustering with sample annotation identified the different origins of B cells. (**B**) Cell type annotation classified B cells into pre-B cells, immature B cells, plasma cells, multi-lymphoid progenitor cells, and lymphoid cells. (**C**) T-SNE plots profiled typical marker genes in B cells, including CD19, CD24, IGHM, IGHD, TMEM173, and GSDMD. (**D**) The expression levels of typical marker genes were compared by dot plot. **E-H**. Transcriptome features of TMEM173 in B cells from B-ALL patients. B cell clusters were extracted from patients’ BM cells. (**E**) Cell type annotation classified B cells into 5 categories. (**F**) Sample annotation identified B cells from different patients. (**G**) T-SNE plots profiled typical marker genes in B cells, including MME, MZB1, IGLC2, IGHD, TMEM173, and GSDMD. (**H**) The expression levels of typical marker genes were compared by dot plot.

Having revealed the importance of B cells in B-ALL, we sought to explore the expression features of TMEM173 in B cells. B cell clustering identified a difference in cellular compositions between B-ALL patients and healthy donors (Fig. 2A). Of note, a group of B cells independently enriched in B-ALL patients was considered to be pre-B cells (cluster 1, 2) (Fig. 2B, **Supplementary Table 3**). Feature gene analysis showed that pre-B cells were characterized by CD19 and CD24 rather than immunoglobulin heavy constant delta (IGHD) and immunoglobulin heavy constant mu (IGHM), which indicated the immatureness of pre-B cells (Fig. 2C). Additionally, a small portion of pre-B cells expressed low levels of TMEM173 and GSDMD (Fig. 2D). These results suggested that B cells of B-ALL patients underwent transcriptional alterations.

To gain insight into the transcriptome features of B cells in B-ALL, B cell clusters were isolated from B-ALL patients, followed by marker gene extraction (**Supplementary Table 3**). 9 clusters were identified by cell clustering, further classified into pre-B cells (cluster 0, 2, 3), immature B cells (cluster 1, 4, 5), plasma cells (cluster 6), lymphoid cells (cluster 7), and multi-lymphoid progenitor cells (cluster 8) **(**Fig. 2E**)**. However, clusters of the same type were unable to cluster together. Sample annotation implied that the decentralized clustering might result from individual differences between B-ALL patients **(**Fig. 2F**)**. Feature plots were then performed to identify leukemic cells. Of note, SRY-Box transcription factor 4 (SOX4) and cyclin-dependent kinase 6 (CDK6) were enriched in pre-B cell clusters, suggesting that pre-B cells of B-ALL patients were leukemic cells (**Supplementary Fig. 3A**). Leukemic cells highly expressed membrane metalloendopeptidase (MME) and MZB1, which was different from immature B cells labeled by IGHD and IGLC2 (Fig. 2G). Besides, a small fraction of leukemic cells retained the expression of TMEM173 and GSDMD (Fig. 2G). TMEM173 was critical for cell death regulation in tumor cells, including apoptosis, autophagy, pyroptosis, and the like [43]. Pyroptosis was a non-apoptotic cell death dependent on the cleavage of gasdermin proteins [44]. Of note, expression levels of TMEM173 and GSDMD were lower than that of typical marker genes (Fig. 2H). Consequently, leukemic cells down-regulate TMEM173 to evade cell death and anti-tumor immunity.

#### Proliferating pre-B cells expressed low levels of GSDMD during the progression of B-ALL

**Fig. 3:**
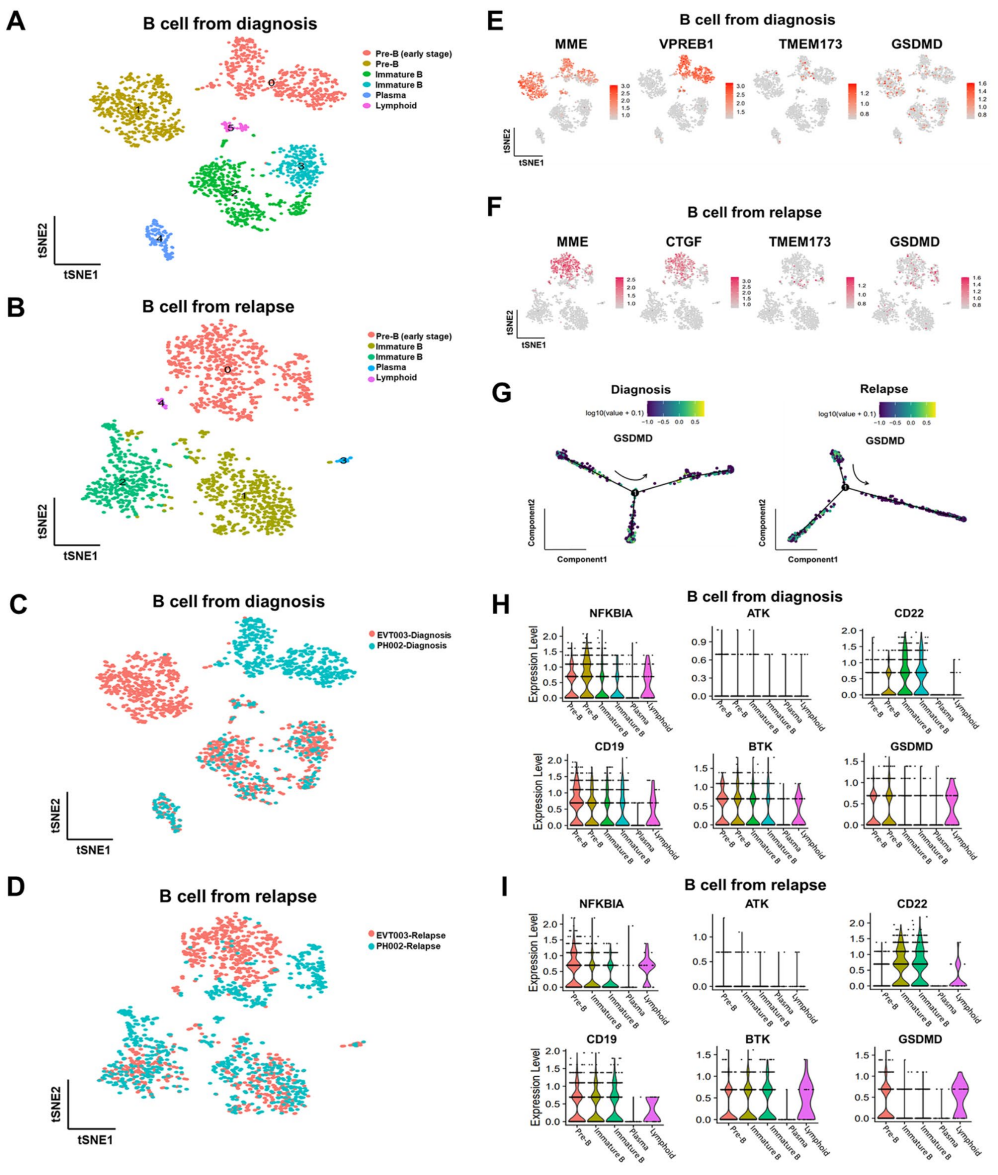
Proliferated pre-B cells expressed GSDMD during the progression of B-ALL. **A, B.** B cell clusters were extracted from initial diagnosis and relapse, respectively. Cell clustering with cell type annotation identified pre-B cells, immature B cells, plasma cells, and lymphoid cells in both diagnosis (**A**) and relapse (**B**). **C, D.** Sample annotation assists in distinguishing B cells from different B-ALL patients at the diagnosis (**C**) and relapse (**D**) stages. **E.** T-SNE plots profiled the expression of MME, VPREB1, TMEM173, and GSDMD in B cells from diagnosis. **F.** T-SNE plots exhibited the expression of MME, CTGS, TMEM173, and GSDMD in B cells from relapse. **G.** Pseudo-time analysis revealed the expression of GSDMD with the developmental trajectory of B cells in both the initial diagnosis and relapse stage. The arrows represented the direction of GSDMD attenuation. **H, I.** Comparison of specific genes (NFKB1A, ATK, CD22, CD19, and BTK) with GSDMD in diagnosis (**H**) and relapse (**I**) by violin plots.

Given that tumor progression could interfere with cell transcriptional profiles, B cell populations were extracted from different stages of B-ALL patients. Clustering analysis revealed the different cell compositions in diagnosis and relapse (**Supplementary Fig. 3B**). Subsequently, B cells from different stages were merged respectively and screened for marker genes (**Supplementary Table 4)**. Cell clustering identified that B cell populations were classified into 4 categories in diagnosis and recurrent stages, which included pre-B cells, immature B cells, plasma cells, and progenitor cells **(**Fig. 3A, B**)**. Moreover, pre-B cells were individually variable at initial diagnosis but were similar at recurrence (Fig. 3C, D). We further explored the differential marker genes of pre-B cells with B-ALL progression. In addition to MME, V-set Pre-B cell surrogate light chain 1 (VPREB1) was increased in diagnosis stage, while cellular communication network factor 2 (CCN2, or CTGF) was increased in recurrence (Fig. 3E, F). Since CTGF was related to the regulation of cell proliferation and adhesion [45], these results suggested that leukemic cells enhanced proliferation viability and invasiveness with B-ALL progression.

We also investigated the expression features of TMEM173 and GSDMD in pre-B cells. Although TMEM173 and GSDMD were consistently expressed at low levels, some pre-B cells restrained GSDMD expression during the progression of B-ALL (Fig. 3E, F**)**. The expression dynamics of GSDMD were then determined by pseudo-time analysis. Specific marker genes were used to identify developmental stages of B cells, including MME and membrane spanning 4-domains A1 (MS4A1, or CD20) (**Supplementary Fig. 4A, B**). Of note, GSDMD expression was gradually decreased with the maturation of B cells, especially in recurrence (Fig. 3G**)**.

To further investigate the proliferative properties of GSDMD-positive pre-B cells, expression levels of GSDMD and proliferation-related genes were illustrated using violin plots. As a result, nuclear factor kappa-B (NF-κB), CD19, and Bruton’s tyrosine kinase (BTK), rather than CD22 and AKT serine/threonine kinase 1 (AKT1), were highly expressed in GSDMD-positive pre-B cells during the progression of B-ALL (Fig. 3H, I). Therefore, these findings imply the potential role of GSDMD in regulating the cell growth of pre-B cells in B-ALL.

### Transcriptome features of TMEM173 in NK cells and DCs

In addition to B cells, TMEM173 expression was decreased in NK cells and DCs from the BM of B-ALL patients. Accumulating evidence demonstrated that the expression of TMEM173 in immune cells was critical for anti-tumor immune response and TME remodeling [46]. Thus, we attempted to explore the transcriptome features of TMEM173 in NK cells and DCs.

#### Cytotoxic activity of NK cells expressed TMEM173 in the BM of B-ALL patients

**Fig. 4:**
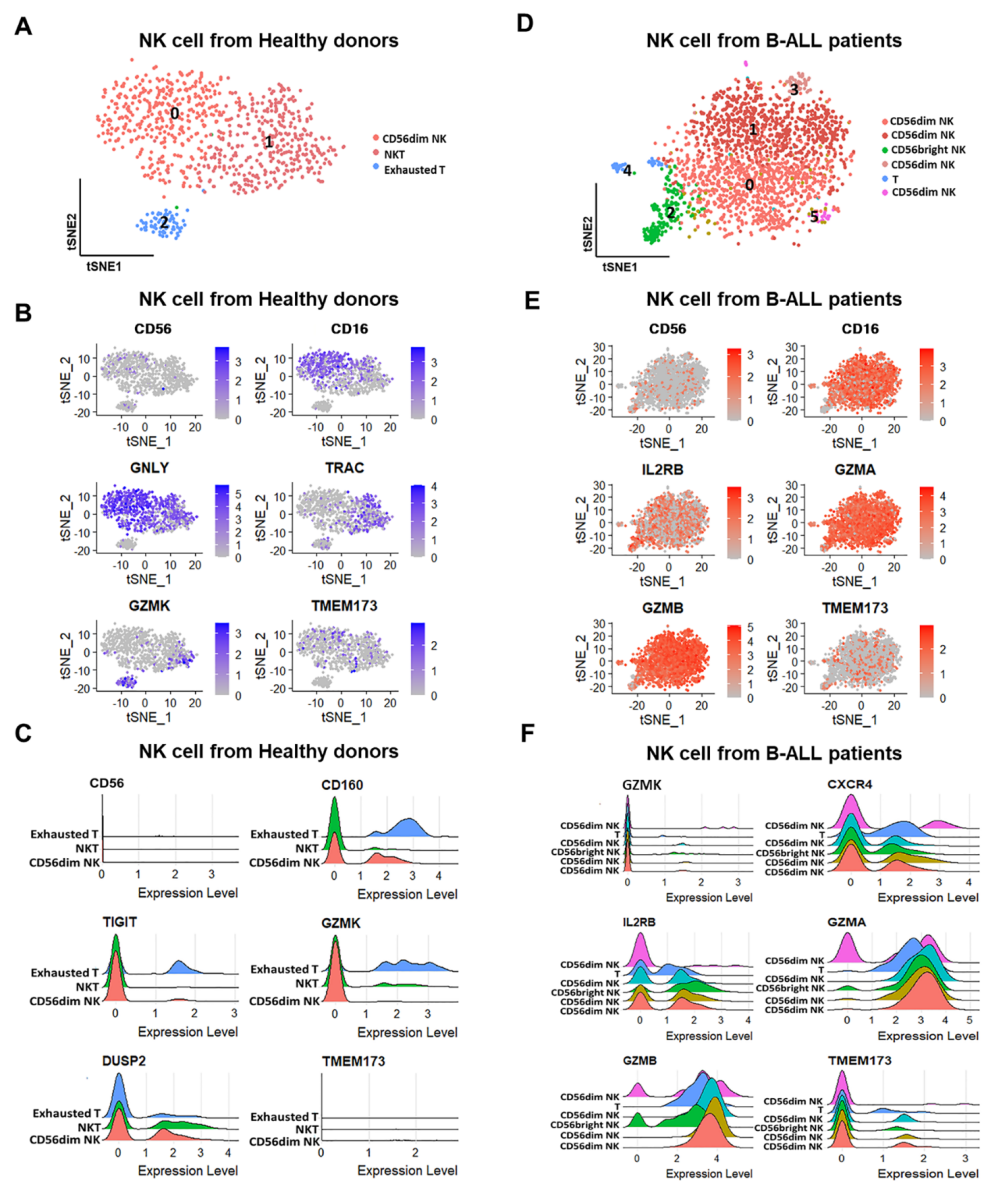
TMEM173 expression was associated with cytotoxic activity of NK cells in the BM of B-ALL. **A-C.** Transcriptional features of TMEM173 in NK cells from the BM of healthy donors. NK cells were isolated from healthy BM cells. (**A**) Clustering identified 3 clusters of NK cells. (**B**) T-SNE plots profiled the expression of typical marker genes (CD56, CD16, GNLY, TRAC, and GZMK) and TMEM173. (**C**) Ridge plots compared the expression levels of TMEM173 with specific biomarkers, including CD56, CD160, TIGIT, GZMK, and DUSP2. **D-F**. Transcriptional features of TMEM173 in NK cells from B-ALL patients. NK cells were isolated from patients’ BM cells. (**D**) Cell clustering identified 6 clusters of NK cells. (**E**) T-SNE plots profiled the expression of typical marker genes (CD56, CD16, IL2RB, GZMA, and GZMB) and TMEM173. (**F**) Ridge plots compared the expression levels of TMEM173 with activation biomarkers, including CD56, CXCR4, IL2RB, GZMA, and GZMB.

We extracted NK cell clusters from healthy donors to identify the transcriptome features of NK cells in a healthy BM environment. Cluster analysis showed that 3 clusters with different marker genes were identified in healthy donors (**Supplementary Table 5**). Granulysin (GNLY), CD56, CD16, T cell receptor alpha constant (TRAC), and granzymes were regarded as the typical marker genes, which facilitated the identification of CD56dim NK cells (GNLY^+^CD56^−^CD16^+^) and NKT cells (GNLY^+^TRAC^+^) (Fig. 4A, B). Additionally, t-SNE plots revealed a wide distribution of TMEM173 in NK cells (Fig. 4B). Given the functions of TMEM173 in immune cell activation [46], we further applied ridge plots to clarify the activation state of NK cells. Notably, inhibitory molecules were increased in NK cells and exhausted T cells, including CD160, dual specificity phosphatase 2 (DUSP2), TIGHT, and granzyme K (GZMK) (Fig. 4C). These findings indicated that NK cells were resting in the BM of healthy donors. Consistently, expression levels of TMEM173 were distinctly lower than that of inhibitory molecules, consistent with the resting state of NK cells (Fig. 4C).

Next, we investigated the composition and expression feature of NK cells in B-ALL patients. As shown in Fig. 4D, CD56dim and CD56bright NK cells were identified in B-ALL patients. CD56dim NK cells expressed CD16, granzyme A (GZMA), and granzyme B (GZMB). CD56bright NK cells increased CD56 and interleukin 2 receptor subunit beta (IL2RB) (Fig. 4E). In particular, expression of TMEM173 was widespread in NK cells, which promoted us to explore the correlation between TMEM173 expression and functional changes of NK cells. Ridge plots illustrated that cytokine receptor (C-X-C motif chemokine receptor 4 (CXCR4), IL2RB) and cytotoxic granzymes (GZMA, GZMB) were increased with TMEM173 in CD56dim NK cells (Fig. 4F). Herein, TMEM173 expression is associated with the cytotoxic activity of NK cells in B-ALL.

#### TMEM173 were related to functional activation of DCs in B-ALL patients

**Fig. 5:**
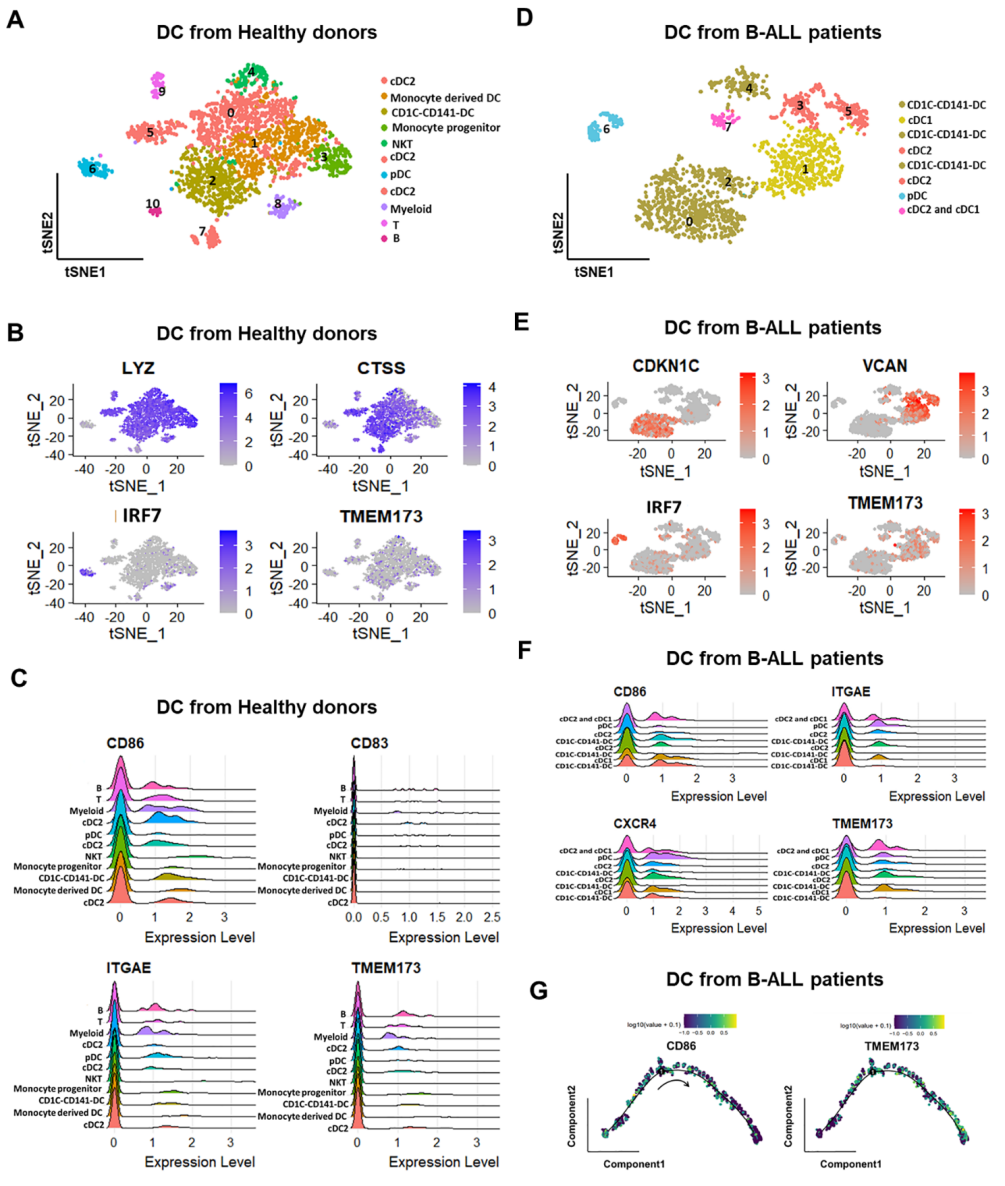
TMEM173 expression was related to functional activation of DCs in the BM of B-ALL. **A-C.** Transcriptional features of TMEM173 in DCs from healthy donors. DCs were isolated from healthy BM cells. (**A**) Clustering identified 11 clusters of DCs. (**B**) T-SNE plots profiled the expression of typical marker genes (LYZ, CTSS, and IRF7) and TMEM173. (**C)** Ridge plots compared the expression levels of TMEM173 with immuno-activating markers (CD86, CD83, and ITGAE). **D-G**. Transcriptional features of TMEM173 in DCs from B-ALL patients. DCs were isolated from patients’ BM cells. (**D**) Clustering identified 8 clusters of DCs. (**E**) T-SNE plots profiled the expression of typical marker genes (CDKN1C, VCAN, and IRF7) and TMEM173. (**F**) Ridge plots compared the expression levels of TMEM173 with immuno-activating markers (CD86, ITGAE, and CXCR4). (**G)** Pseudo-time analysis profiled the expression trajectory of TMEM173 during the development of DCs. The arrow represented the direction of development.

As an important member of immune regulation, DC is essential for the anti-tumor immune response [47]. DCs were then isolated from BM cells and subjected to feature analysis. Based on specific marker genes, DCs were separated into different populations (**Supplementary Table 6**). In the BM of healthy donors, type II classical DCs (cDC2s, cluster 0, 5, 7), monocyte-derived DCs (cluster 1), CD1c^−^CD141^−^DCs (cluster 2), and plasmacytoid DCs (pDCs, cluster 6) were the major populations of DCs **(**Fig. 5A**)**. A small proportion of NKT cells (cluster 4), T cells (cluster 9) and B cells (cluster 10) were accompanied with DCs, implying the recruiting functions of DCs (Fig. 5A). To evaluate the killing activity of DCs, t-SNE and ridge plots were performed. High levels of lysozyme (LYZ) and cathepsin S (CTSS) were profiled in cDC2s, monocyte-derived DCs, and CD1c^−^CD141^−^DCs **(**Fig. 5B**)**. Interferon regulatory factor 7 (IRF7) was increased in pDCs, which was consistent with the functions of producing type I interferon (IFN) (Fig. 5B). Ridge plots further identified that DCs decreased the expression of activation markers, such as CD86, CD83 and integrin subunit alpha E (ITGAE), suggesting that DCs from healthy donors were in an inactivated state (Fig. 5C). Of note, the expression level of TMEM173 in inactivated DCs was lower than lymphoid cells (Fig. 5C).

Transcriptome features of DCs were also explored in B-ALL. Cell clustering identified 4 populations of DCs in the BM of B-ALL patients, including type I classical DCs (cDC1s), cDC2s, CD1c^−^CD141^−^DCs, and pDCs (Fig. 5D). Compared with healthy donors, the proportion of versican (VCAN)^+^ cDC1s was increased in B-ALL patients (Fig. 5D, E). Besides, the typical marker gene of CD1c^−^CD141^−^DCs turned into cyclin-dependent kinase inhibitor 1 C (CDKN1C) (Fig. 5E). Alterations in subtype compositions and feature genes indicated that the functions of DCs were changed in B-ALL. Besides, t-SNE plots profiled a broad expression of TMEM173 in DCs (Fig. 5E), which promoted us to investigate the activation states of DCs in B-ALL. As shown in Fig. 5F, cell activation markers (CD86 and ITGAE) and chemokine receptor (CXCR4) were elevated in all subtypes of DCs, consistent with the expression of TMEM173. To further explore the dynamic evolution of TMEM173 expression with DC development, we performed a pseudo-time analysis in DCs. As a result, TMEM173 expression was increased with the maturation of DCs (**Supplementary Fig. 5A**, Fig. 5G). Altogether, we suggest that TMEM173 expression is related to the functional activation of DCs in B-ALL.

## Discussion

In this present study, we first explored the transcriptome features of TMEM173 in BM cells of high-risk B-ALL at a single-cell resolution. TMEM173 was differentially expressed in BM cells, of which B cells, NK cells, and DCs were featured with low levels of TMEM173. On the one hand, proliferated pre-B cells featured low levels of TMEM173 and downstream pyroptosis effector GSDMD during the progression of B-ALL. On the other hand, TMEM173 expression was associated with the functional activation of NK cells and DCs. Our findings indicated the potential role of TMEM173 in the anti-tumor therapy of B-ALL.

TMEM173 plays a significant role in tumorigenesis, anti-tumor immune response, and cell death induction. Notably, recent studies demonstrate that TMEM173 is involved in the development of acute leukemia. Activation of TMEM173 depresses the hematopoietic capacity of hematopoietic stem cells (HSCs) and the viability of acute myeloid leukemia (AML) cells [48, 49]. Our results identify the low levels of TMEM173 in malignant B cells, which meet the demands of rapid cell proliferation. In addition to cell viability, various kinds of cell death are under the regulation of TMEM173 [50]. In particular, TMEM173 is involved in pyroptosis, of which the canonical pathway depends on TMEM173-mediated GSDMD cleavage [51, 52]. Inducing pyroptosis in tumor cells has been regarded as an effective strategy for reducing tumor burden [53, 54]. For B-ALL, tumor burden mainly derives from the clonal expansion of malignant B cells [55]. Notably, a portion of pre-B cells restrains the expression of GSDMD with B-ALL progression, indicating the possibility of triggering GSDMD-dependent pyroptosis in leukemic cells. Besides, GSDMD is mainly expressed in pre-B cells with multiplication capacity, represented by high expression of NF-κB, CD19, and BTK. It is well known that activation of NF-κB and the CD19-BTK axis is essential for promoting B cell growth [56, 57]. Herein, targeted activation of TMEM173 in leukemic cells may induce GSDMD-dependent pyroptosis and reduce the tumor burden of B-ALL.

R/R B-ALL is a critical challenge in clinical practice since the prognosis for adult R/R B-ALL remains poor. In a recent study, Tang et al. illustrated that combining cell pyroptosis and immunotherapies significantly enhanced the anti-tumor effects, especially in drug-resistant cases [58]. CAR-T cells targeting the CD19 antigen represent an innovative therapeutic approach for R/R B-ALL [59, 60]. Besides, anti-CD19 CAR-T therapy is demonstrated to induce GSDMD-mediated cell pyroptosis in B cell-derived tumor cells [61]. Given that CD19 is co-expressed with GSDMD in pre-B cells, the combination of anti-CD19 CAR-T cells and TMEM173 agonists will hopefully magnify the efficiency of CAR-T cell therapies in B-ALL.

TME-infiltrating immune cells are the other essential regulators in enhancing the efficacy of anti-tumor therapies [16]. Consistently, our investigations identify an increase of NK cells and DCs in the BM of B-ALL. Activation of NK cells has been reported to improve the therapeutic efficiency through inducting tumor cell death [62–64]. We consistently demonstrate that cytotoxic NK cells of B-ALL were marked by increased TMEM173, which indicated the importance of TMEM173 in activating the anti-tumor functions of NK cells. DCs are the other component in anti-tumor immune response [65]. ScRNA-seq analysis reveals an increase in cDC1s and pDCs with immune activation phenotypes in the BM of B-ALL. Accumulating cDC1s is essential for the T cell-mediated anti-tumor response [66], while pDCs contribute to IFN-α/β secretion [67]. Activation of TMEM173 in cDC1s and pDCs promotes the recruitment and functional activation of DCs in TME [16, 68], further improving clinical outcomes [69]. Accordingly, TMEM173 is consistent with immune activation phenotypes of DCs in B-ALL, suggesting that TMEM173 activation might be associated with enhanced anti-tumor functions of DCs. Therefore, targeted activation of TMEM173 in immune cells is expected to be a feasible strategy for improving therapeutic efficiency and clinical outcomes in B-ALL.

A recent study demonstrates that gene mutations impact the functions of TMEM173 in regulating immune activation [70]. Although the TMEM173 locus is absent in our next-generation sequencing (NGS) panels, Sanger sequencing identifies frameshift mutation in PBMCs of several B-ALL patients. However, the correlation between mutations and expression of TMEM173 could not be assessed due to insufficient samples. Increasing the number of patient samples and targeted sequencing panels is the focus of future research.

Overall, our findings extend the understanding of the transcriptome features of TMEM173 in B-ALL. It is worth noting that TMEM173 agonists exhibit pronounced anti-tumor effects in advanced solid tumors and lymphomas [71]. However, the transcriptome landscape cannot demonstrate the biological function of TMEM173 in B-ALL. More functional experiments will be performed in subsequent research to confirm the importance of TMEM173 in B-ALL.

## Conclusion

In summary, we profile the transcriptional features of TMEM173 in the BM of high-risk B-ALL patients by a scRNA-seq analysis. TMEM173 expression is decreased in proliferated leukemic cells and activated immune cells, where targeted activation of TMEM173 is expected to be a new therapeutic strategy for B-ALL.

## Electronic supplementary material

Below is the link to the electronic supplementary material.

Supplementary Material 1

Supplementary Material 2

## Data Availability

The datasets analyzed during the current study are available in the Gene Expression Omnibus (GEO), (https://www.ncbi.nlm.nih.gov/geo/query/acc.cgi?acc=GSE130116).

## Abbreviations

AKT1: AKT serine/threonine kinase 1
AML: acute myeloid leukemia
B-ALL: B-cell acute lymphoblastic leukemia
BCR: B cell receptor
BM: bone marrow
BTK: bruton’s tyrosine kinase
CAR-T: chimeric antigen receptor T
CCN2: cellular communication network factor 2
cDC1s: classical DCs
CDK6: cyclin-dependent kinase 6
CDKN1C: cyclin dependent kinase inhibitor 1 C
CR: complete remission
CTSS: cathepsin S
DCs: dendritic cells
CXCR4: C-X-C motif chemokine receptor 4
DEGs: differentially expressed genes
DNTT: DNA nucleotidylexotransferase
DUSP2: dual specificity phosphatase 2
GEO: Gene Expression Omnibus
GNLY: granulysin
GSDMD: gasdermin D
GZMA: granzyme A
GZMB: granzyme B
GZMK: granzyme K
HSCs: hematopoietic stem cells
IFN: interferon
IGHA1: immunoglobulin heavy constant alpha-1
IGHD: immunoglobulin heavy constant Delta
IGHG1: immunoglobulin heavy constant gamma-1
IGHG3: immunoglobulin heavy constant gamma-3
IGHM: immunoglobulin heavy constant Mu
IGKC: immunoglobulin kappa constant
IGLC2: immunoglobulin lambda constant 2
IGLC3: immunoglobulin lambda constant 3
IL2RB: interleukin 2 receptor subunit beta
IRF7: interferon regulatory factor 7
ITGAE: integrin subunit alpha E
JCHAIN: joining chain of multimeric IgA and IgM
LYZ: lysozyme
MME: membrane metalloendopeptidase
MS4A1: membrane spanning 4-domains A1
MZB1: marginal zone B and B1
NF-κB: nuclear factor kappa-B
NGS: Next-generation sequencing
NK cells: natural killer cells
PBMCs: peripheral blood mononuclear cells
PC: principal component
PCA: principal component analysis
pDCs: plasmacytoid DCs
pre-B cells: precursor B cells
PVDF: polyvinylidene fluoride
qRT-PCR: quantitative real-time PCR
R/R: refractory /relapse
ScRNA-seq: single-cell RNA sequencing
SD: standard deviation
SOX4: SRY-Box transcription factor 4
TBST: Tris-buffered saline and Tween 20
TRAC: T cell receptor alpha constant
TME: tissue-specific tumor microenvironment
TMEM173/STING: stimulator of interferon genes
T-SNE: t-distributed stochastic neighbor embedding
VCAN: versican
VPREB1: V-set pre-B cell surrogate light chain 1
WB: Western blotting
WHO: World Health Organization.
