# Supplementary material for: Single-cell transcriptome analysis profiles the expression features of TMEM173 in BM cells of high-risk B-cell acute lymphoblastic leukemia
Source: BMC Cancer. 2023 Apr 24;23:372. doi: 10.1186/s12885-023-10830-5 (PMC10123968; doi:10.1186/s12885-023-10830-5)
Supplement: Supplementary file 2 — Supplementary Material 2 [file 12885_2023_10830_MOESM2_ESM.docx]

**Supplementary Fig. 1** Relative mRNA expression and gene sequence of TMEM173 in PBMCs.

**
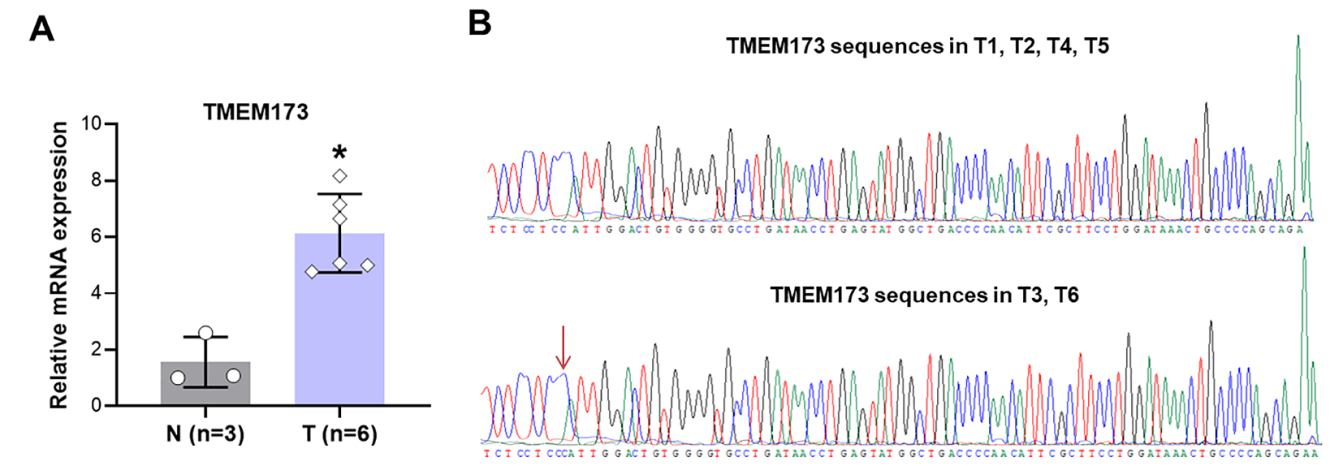
**

**A, B.** Total RNA was extracted from PBMCs of each sample (N1-N3=healthy donors; T1-T6=B-ALL patients), followed by genomic DNA wiper and reverse transcription. (**A**) PCR showed that mRNA levels of TMEM173 were increased in PBMCs from B-ALL patients (Reference gene: β-actin). The statistical significance was determined by the unpaired two-tailed T-test. ^*^p<0.05. (**B**) TMEM173 sequences in different patient samples were detected by Sanger sequences and profiled by peak plots. The inserted base was marked by a red arrow.

**Supplementary Fig. 2** Cell clustering and marker genes of BM cells.


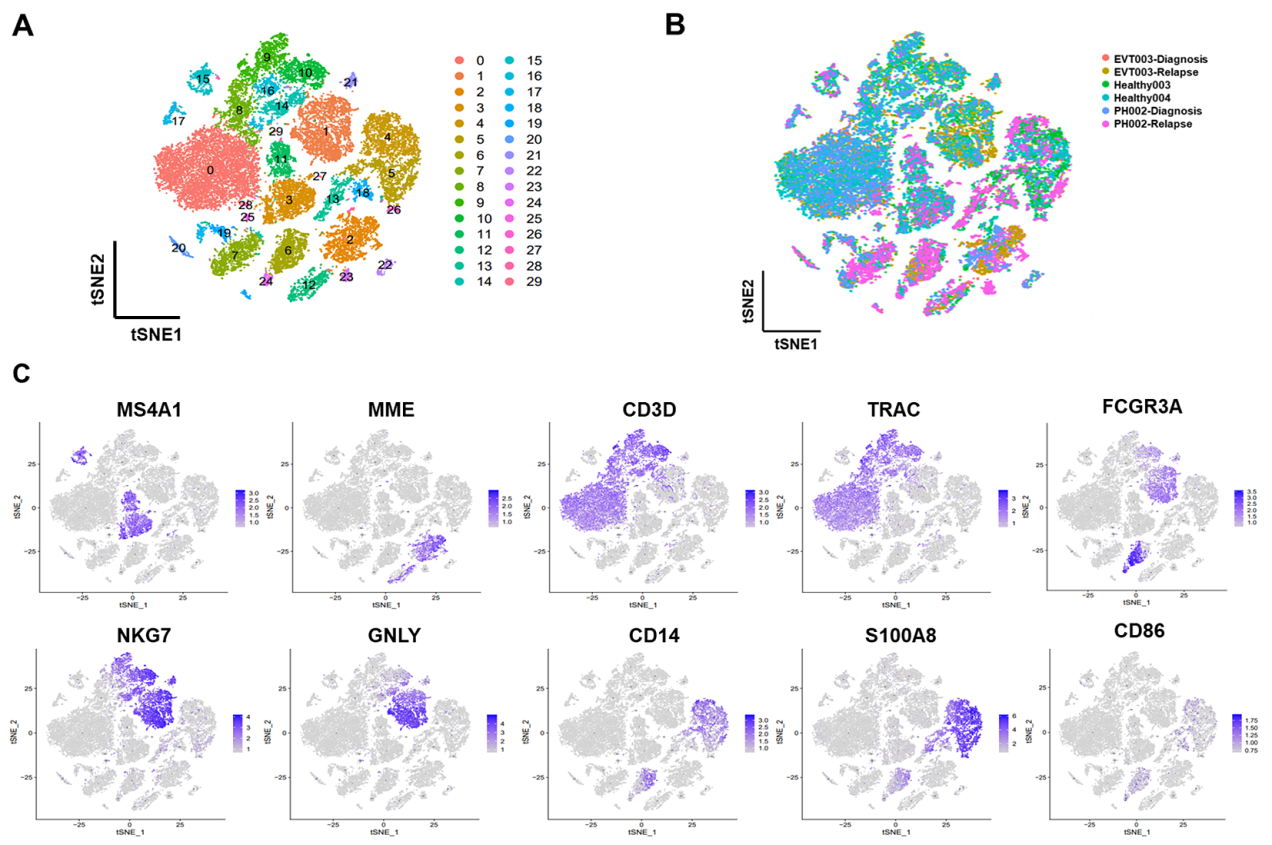


**A-C.** ScRNA-seq data of 2 healthy donors and 2 B-ALL patients (both diagnosis and relapse) were merged. **(A)** Cell clustering identified 30 cell clusters in BM cells. **(B)** T-SNE plot identified the origins of BM cells from different individual. (**C**) T-SNE plots revealed typical marker genes of different cell types, including B cell (MS4A1, MME), T cell (CD3D, TRAC), NK cell (NKG7, GNLY), monocyte (CD14, S100A8), and DC (FCGR3A, CD86).

**Supplementary Fig. 3** Feature analysis of B cells from diagnostic and relapse B-ALL patients.

**
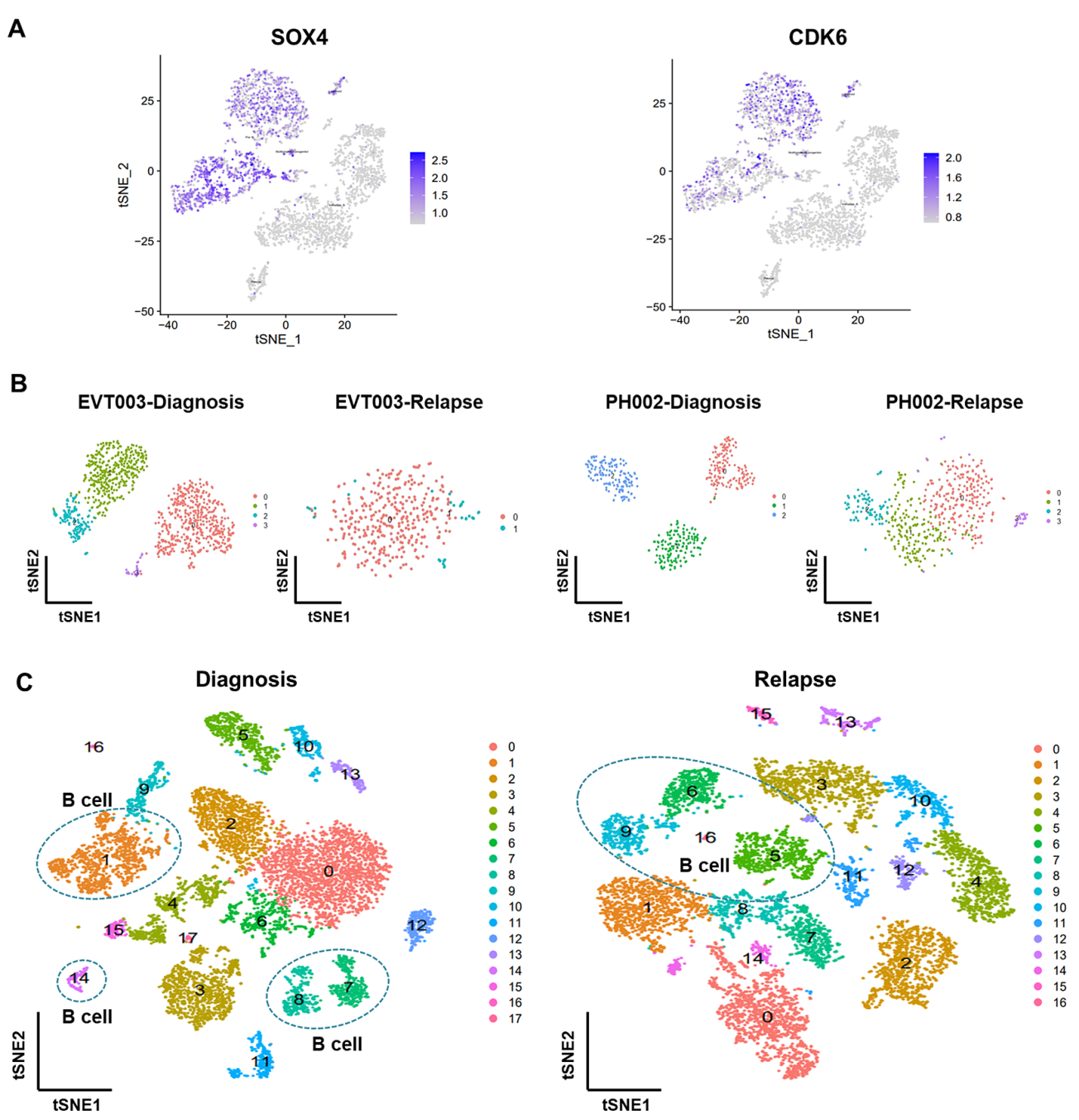
**

**A.** B cell clusters were extracted from BM cells of B-ALL patients. After cell clustering, leukemic cells were labeled by SOX4 and CDK6 in T-SNE plots. **B.** Cell clustering of B cells from EVT003 and PH002 patients at different stages.


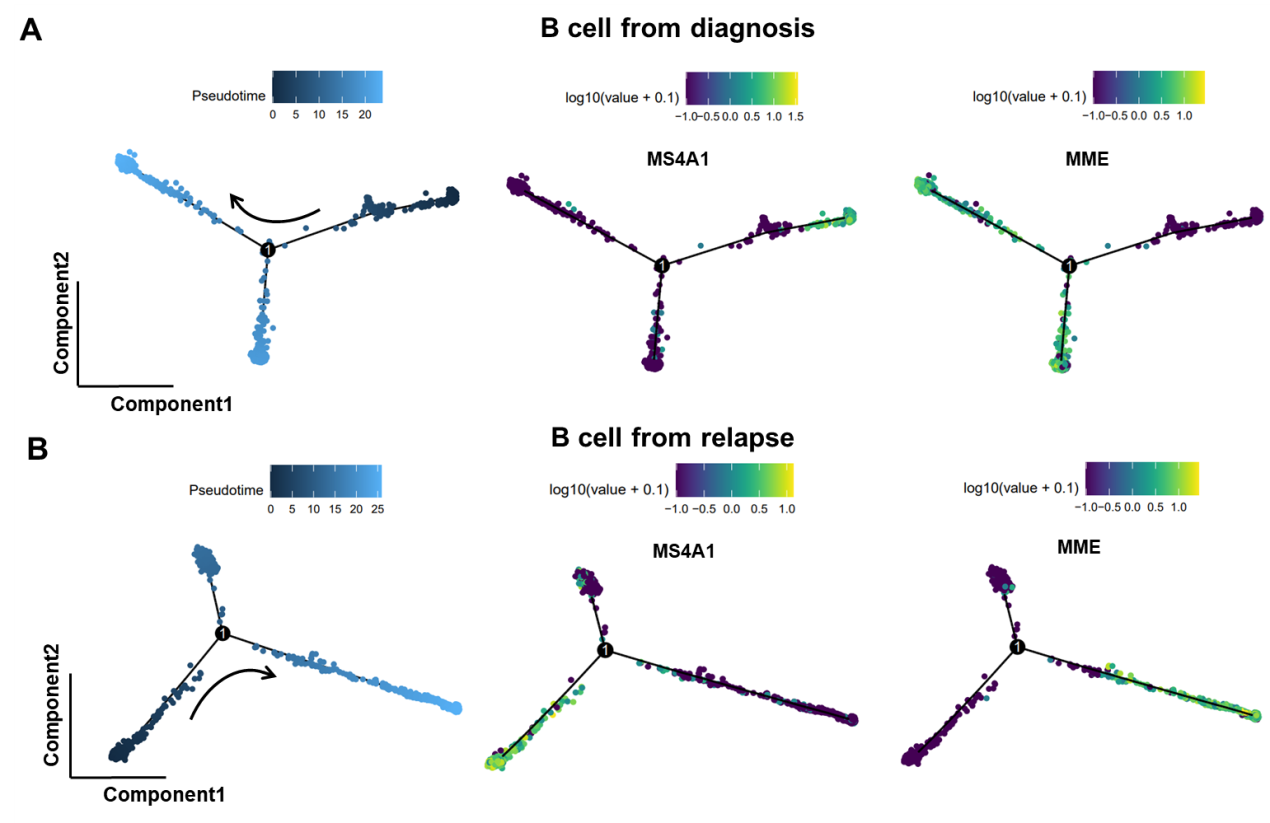
**Supplementary Fig. 4** Developmental trajectories and gene expression trajectories of B cells from B-ALL patients.

**A, B.** Pseudo-time analysis revealed the developmental trajectories of B cells, accompanying by the expression trajectories of MS4A1 and MME in B cells from B-ALL patients at the diagnosis (**A**) and the relapse (**B**) stages. The direction of arrows represented the stage of development.

**Supplementary Fig. 5** Developmental trajectories and gene expression trajectories of DCs from B-ALL patients.


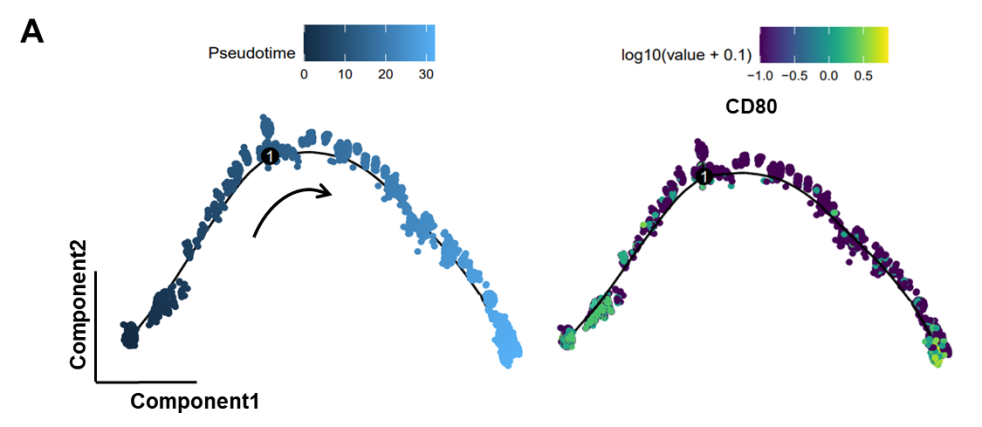


**A.** Pseudo-time analysis revealed the developmental trajectories of DCs, accompanying by the expression trajectories of CD80 in DCs from B-ALL patients. The direction of arrow represented the stage of development.

**Supplementary Fig. 6** The original images of WB from 3 replicate experiments.

Repeat 1 TMEM173 (40kdDa) GAPDH (37kDa)


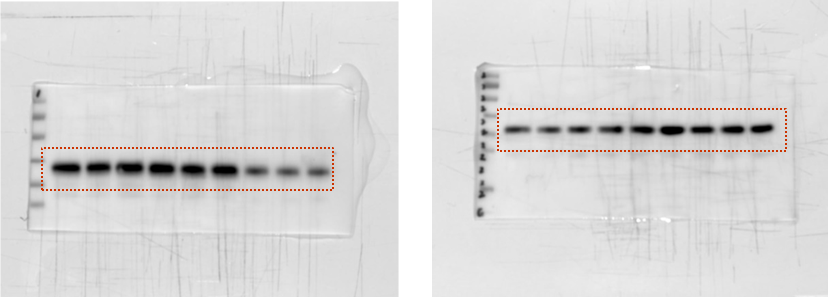


Repeat 2 TMEM173 (40kdDa) GAPDH (37kDa)


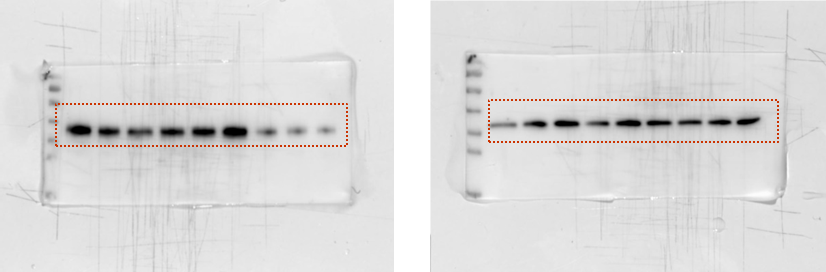


Repeat 3 TMEM173 (40kdDa) GAPDH (37kDa)


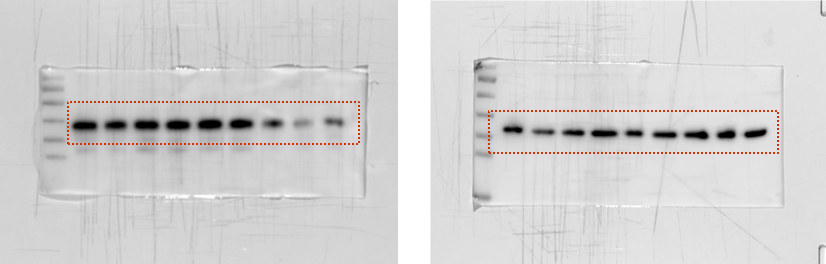


The original images of WB from 3 replicate experiments. The selected area within the dashed line from the first repeat experiment was presented in **Fig. 1B**.
